# Improved Establishment of Embryonic Stem (ES) Cell Lines from the Chinese Kunming Mice by Hybridization with 129 Mice

**DOI:** 10.3390/ijms15033389

**Published:** 2014-02-25

**Authors:** Shumin Yu, Xingrong Yan, Huanhuan Liu, Xin Cai, Suizhong Cao, Liuhong Shen, Zhicai Zuo, Junliang Deng, Xiaoping Ma, Ya Wang, Zhihua Ren

**Affiliations:** 1 College of Veterinary Medicine, Sichuan Agricultural University, Xinkang Road 46^#^, Yucheng District, Ya’an 625014, Sichuan, China; E-Mails: yayushumin@163.com (S.Y.); liuhuanhd@163.com (H.L.); suizhongcao@126.com (S.C.); shenlh@sicau.edu.cn (L.S.); dengjl213@126.com (J.D.); mxp886@sina.com (X.M.); wangyayang@126.com (Y.W.); zhihua_ren@126.com (Z.R.); 2 Life Science College, North-West University, Xi’an 710069, Shaanxi, China; E-Mail: xingrongyan2007@126.com; 3 School of Life Science and Engineering, Southwest University of Science and Technology, Mianyang 621010, Sichuan, China; E-Mail: caixin2323@126.com

**Keywords:** Chinese Kunming mouse (*Mus musculus* Km), 129/Sv mouse, hybrid embryos, embryonic stem (ES) cells

## Abstract

Chinese Kunming mice (*Mus musculus* Km), widely used as laboratory animals throughout China, remain very refractory for embryonic stem (ES) cell isolation. The present study was aimed to evaluate the effects of hybridization with 129/Sv mice, and culture media containing fetal bovine serum (FBS) or Knockout serum replacement (KSR) on ES cell isolation from Kunming mice. The results demonstrated that ES cells had been effectively isolated from the hybrid embryos of Kunming and 129/Sv mice using all three media containing 15% FBS, 15% KSR and their mixture of 14% KSR and 1% FBS, individually. These isolated ES cells had maintained *in vitro* undifferentiated for a long time, exhibiting all features specific for mouse ES cells. In addition, the rates of ES cell isolation in the medium containing 14% KSR and 1% FBS, was 46.67% and significantly higher than those in another two media containing only FBS or KSR (*p* < 0.05). Contrarily, no ES cell line had been established from Kunming mouse inbred embryos using the same protocols. These results suggested that ES cells with long-term self-renewal ability could be efficiently generated from hybrid embryos of Kunming and 129/Sv mice, and a small volume of FBS was necessary to isolate ES cells in the KSR medium when embryos and early ES cells cultured.

## 1. Introduction

Mouse Embryonic stem (mES) cells, which are pluripotential cells from early pre-implantation embryos and have the ability to generate all somatic cells and functional gametes [1,2], are used to explore expression and function of genes *in vivo* by genetic modification [3]. Since Evans *et al*. (1981) first established mES cell lines derived from 129/SvE mice [1], mES cells had been generated from other mouse strains but not the 129 mouse strain, by various combination of feeder cells, fetal bovine serum (FBS)/serum replacement (SR) [4–10], high concentration of leukemia inhibitory factor (LIF) [4,7,9,11], and especially some small-molecular compound including adrenocorticotropic hormone (ACTH) [12,13], pluripotin [14], BMP4 [15] and three inhibitors against signaling pathways of FGFR, MEK and GSK3 [7,16,17].

The Chinese Kunming mouse strain (*Mus musculus* Km, KM), a outbreed mouse strain originating from the Swiss albino mouse, are widely used in pharmacology and genetically related studies throughout China. It exhibits many advantages such as high disease resistance, large and frequent litters and rapid growth rates. Although Peng *et al*. (2013) recently established the germline-competent ES cell lines with long-term self-renewal ability from KM inbred mice in the N2B27 medium supple-mented with PD0325901 and CHIR99021 [18], it remains very refractory to isolate ES cells from this mouse strain under the condition of feeder cells, LIF, FBS or SR. Previous researchers demonstrated that ES cells could be easily isolated from hybrid embryos of 129 mice and the recalcitrant mice DDK and NOD [19,20], and the 129 mice are acknowledged to be the most conducive for ES cell isolation among all mouse strains [21–23]. Moreover, we had established the parthenogenetical ES cell lines from the hybrid offspring oocytes of Kunming and 129/Sv mice using feeder cells, LIF, SR and FBS [24]. Therefore, we investigated effects of hybridization with 129/Sv male mice on ES cell isolation from KM mice using *in vivo* fertilized embryos, in order to explore the genetic/epigenetic mechanism of KM mice which hamper ES cell isolation, and apply the mouse strain for targeted genetic manipulation.

## 2. Results

### 2.1. Effect of FBS and KSR on Derivation of ES Cell from Hybrid Blastocysts

Hybrid embryos for ES cell isolation, which were produced from the pregnant Kunming females mated with 129/Sv males, were cultured on the inactivated MEF feeder layers in the ES media supplemented with 15% Knockout Serum Repacement (KSR), 1% FBS + 14% KSR and 15% FBS, individually. As shown in Table 1, it took significantly longer to achieve embryo attachment in the 15% KSR medium, in which the number of attached embryos was significantly lower than that in another two media. All primary ES cells produced from the picked ICM outgrowths, persisted the undifferentiated state and generated the ES cell lines in the two media containing 15% KSR and the mixture of 1% FBS + 14% KSR. By contrast, only a small amount of ICM outgrowths and primary ES cells further formed ES cell clones due to death or differentiation in the medium containing 15% FBS, although embryos attached to the feeder layers as efficiently as that in the medium containing 14% KSR + 1% FBS (Table 2). Finally, ES cell lines had been established in the medium containing 14% KSR and 1% FBS with the higher efficiency of 46.67%, compared with those in another two media (Table 2).

**Figure 1.**
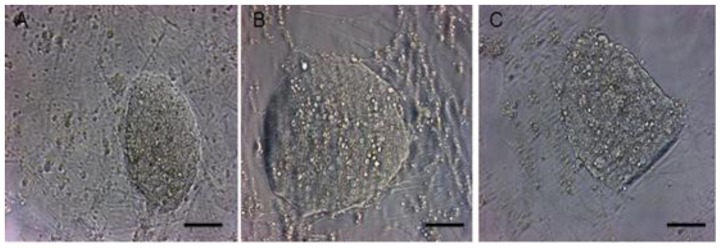
ES cell clone shapes cultured for 7 days in the media containing 15% KSR (**A**); 14% KSR + 1% FBS (**B**); 15% FBS (**C**) when single ES cells were plated. Scale bar = 150 μm.

In addition, when individually plated in the 96-well plates, 14.2% of single ES cells formed cell clones in the 15% KSR medium, which was significantly lower than those in another two media (Table 3). However, ES cell clones in the two media containing KSR (Figure 1A,B), maintained morphologically undifferentiated for a longer time, and ES cell clones in the 15% FBS medium (Figure 1C) exhibited morphologically the aging signs with many dark granules. These results suggested that KSR was preferable to FBS for culturing ES cells, and recombined supplement with KSR and a small amount of FBS contributed to improvement of ES cell isolation when embryos and primary ES cells were cultured.

### 2.2. Effect of Genetic Background on ES Cell Derivation

We had efficiently established several ES cell lines from hybrid embryos of Kunming and 129/Sv mice as shown in Table 2. These ES cells derived from hybrid embryos, usually formed a great number of ES cell clones in the plates with 3.5 cm diameter after the second trypsinization, with the long-term self-renewal. We attempted to isolate ES cells from inbred blastocysts of Kunming mice, but the results demonstrated that under the same condition, the vast majority of picked ICM outgrowths and primary ES cells could not further form ES cell clone after dissociation even though embryos could normally attach and form ICM outgrowths as the hybrid blastocysts. Furthermore, we had increased LIF concentration to 100 ng/mL in the media, which had not significantly promoted *in vitro* propagation of ES cells. As the best results, ES cells derived from only two inbred blastocysts had been maintained in the four-well plates until the seventh passage when ES cell clone disappeared, and ES cell clones gradually decreased due to death or differentiation after being trypsinized. Consequently, no ES cell line had been established from 391 Kunming mouse inbred blastocysts (Table 4).

### 2.3. Characterization of mES Cells

Microsatellite marker-primed PCR results summarized in Table 5, demonstrated that at all examined microsatellite loci except D14Mit113, the tested mES cells exhibited the identical PCR products to the F_1_ hybrid embryos from the Kunming females and 129/Sv males. Moreover, the mES cells and the F_1_ embryos contained the identical PCR products to the Kunming females at the microsatellite loci D1Mit15, D2Mit30, D11Mit4, D11Mit167, D12Mit56 and D14Mit129, and to the 129/Sv males at the microsatellite loci D2Mit30, D11Mit4, D11Mit167, D14Mit129, and D17Mit36, respectively. The results could verify that the tested mES cells contain genetic background of the Kunming strain and 129/Sv strain.

**Figure 2.**
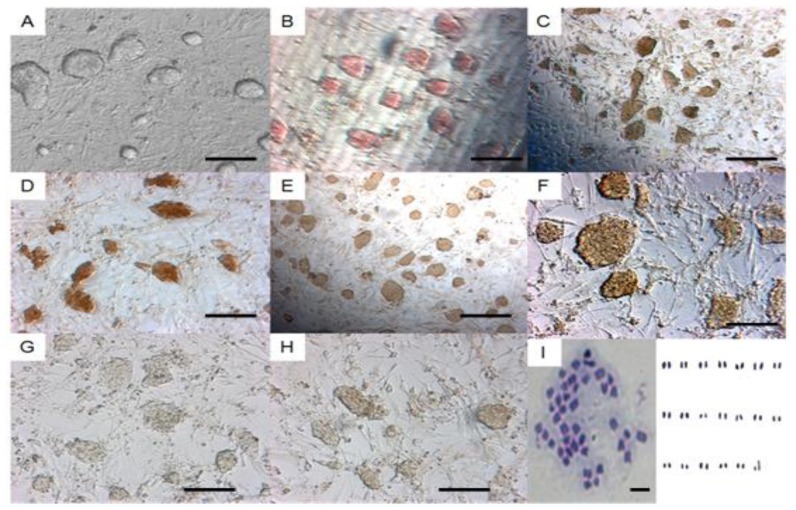
Karyotypes and immunohistochemical characterization of the isolated ES cells. (**A**) the ES cell clones; The ES cells were positive for AKP staining (**B**); telomerase (**C**); Oct4 (**D**); Nanog (**E**) and SSEA-1 (**F**); but negative for SSEA-3 (**G**) and SSEA-4 (**H**) while the ES cells had 20 pairs of chromosomes with XY sex chromosome (**I**, 400×). Scale bar = 100 μm.

**Figure 3.**
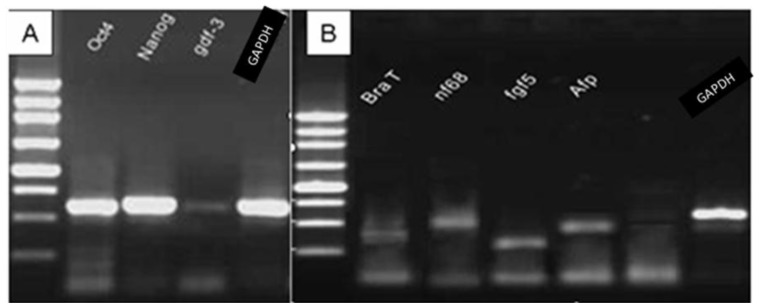
RT-PCR analysis of the expressed marker molecules in the ES cells and the specific genes for three germ layers in the ES cell-derived embryoid bodies. (**A**) dictated the ES cells expressed *Oct*4, *Nanog* and *Gdf*-3; and (**B**) dictated the embryoid bodies (EBs) expressed these specific genes: *BraT* for mesoderm, *Nf-*68 and *Fgf-*5 for ectoderm, *Afp* for endoderm, and *GAPDH* as the positive control.

During the extended culture, the novel ES cells produced from hybrid embryos, always formed morphologically compacted cell clones (Figure 2A) in the presence of MEF feeder layer and LIF, and most of the checked cells (89.5% at the 22th passage *vs*. 75.0% at the 33th passage) maintained diploid karyotypes with 19 pairs of autosomes and XY chromosomes (Figure 2I). These cells were positively stained for AKP (Figure 2B), and by immunohistochemical staining for telomerase (Figure 2C), Oct4 (Figure 2D), Nanog (Figure 2E) and SSEA-1 (Figure 2F), but negatively by immunohistochemical staining for SSEA-3 (Figure 2G) and SSEA-4 (Figure 2H). In addition, RT-PCR analysis showed that the tested ES cells positively expressed *Oct*4, *Nanog* and *Gdf-*3 (Figure 3A), while *Gdf-*3 expression level was relatively lower compared with those of *Oct*4 and *Nanog* (Figure 3A).

**Figure 4.**
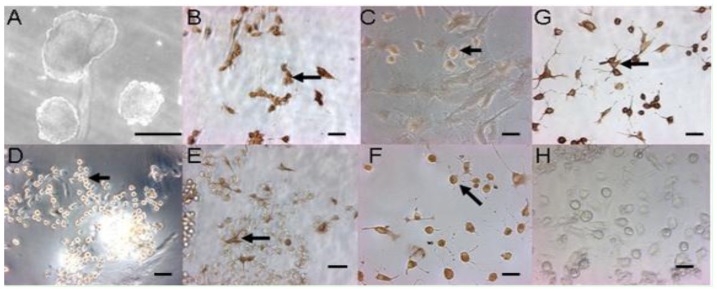
Immunohistochemical analysis of the differentiated cell produced from the ES cell-derived embryoid bodies. The ES cell clumps formed the EBs (**A**) in the leukemia inhibitory factor (LIF)- and feeder- free condition by suspension; and differentiated into cardiomyocytes positive for α-Actin (**B**) and osteoblasts (**C**) positive for Alizarin Red staining from the mesoderm, hepatic cells (**D**) positive for AFP from the endoderm, and neurocytes positive for Nestin (**E**); GFAP (**F**) and β-tubulin III (**G**) from the ectoderm; (**H**) was stained only by the secondary antibody as negative control. Scale bar = 50 μm. In (**B**–**G**), what the arrows indicated were the positive cells stained for the primary antibodies or the dye Alizarin Red, respectively. The arrows in Figure **B**–**G**, indicated the differentiated cells mentioned above.

**Figure 5.**
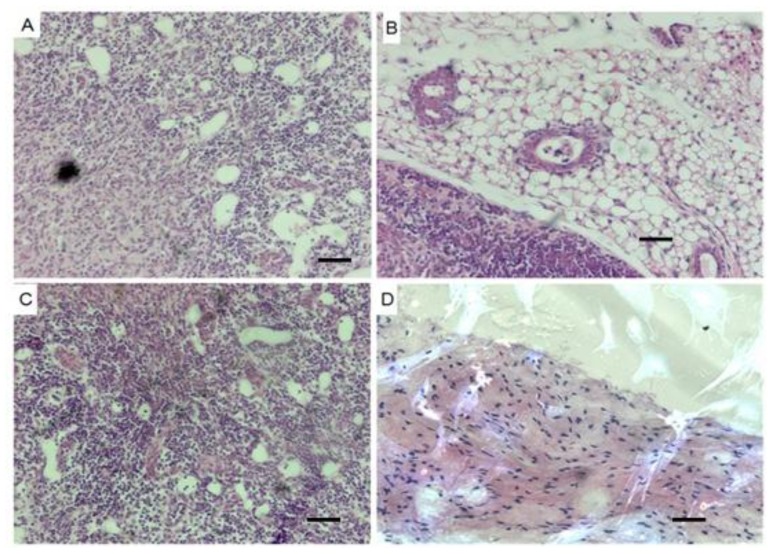
Histological Section of teratomas derived from the ES cells (H/E). Teratoma sections showed various types of tissue structures resembling lymphatic tissues (**A**,**C**); blood tube (**A**); epithelium (**B**); fat tissue (**B**); gland tissue (**B**); muscle (**B**,**C**); connective tissue (**D**). Scale bar = 100 μm.

After removing feeder layer and LIF, ES cell clumps in suspension formed embryoid bodies (EBs) (Figure 4A) in the 15% FBS medium, which differentiated morphologically into various types of cells. These EB-derived cells immunohistochemically presented the specific antigen phenotypes of differentiated cells derived from mesoderm (Figure 4B,C), endoderm (Figure 4D) and neuroectoderm (Figure 4E–G), while EBs expressed the specific marker genes *Fgf*-5 and *Nf*-68 for ectoderm, *BraT* for mesoderm and *Afp* for endoderm (Figure 3B). In addition, the ES cell suspension was subcutaneously injected into nude mice and formed teratomas, the sections of which demonstrated such resembling tissue structure as lymphatic tissues (Figure 5A,C), blood tube (Figure 5A), epithelium (Figure 5B), fat tissue (Figure 5B), gland tissue (Figure 5B), muscle (Figure 5B,C) and connective tissue (Figure 5D).

Together, these results suggested that the isolated ES cells could permanently retain undifferentiated and differentiate into various types of cells from three germ layers *in vitro* or *in vivo*.

## 3. Discussion

Here, we have efficiently isolated ES cells from hybrid embryos of Kunming and 129/Sv mice. The established ES cell lines contained the genetic background of Kunming and 129/Sv mice by microsatellite marker-primed PCR analysis, and shared all features of mES cells including normal karyotypes, high activity of AKP and telomerase, SSEAs antigen profile, differentiated potential into various types of cells from three germ layers *in vitro* or *in vivo*, and expression of two key transcription factors of pluripotency, namely Oct4 and Nanog [25,26]. Moreover, RT-PCR results demonstrated in the isolated ES cells that the gene *Gdf-*3 had been transcribed in a relatively lower level compared with mRNA levels of the genes *Oct*4 and *Nanog*, which accorded with the finding that low expression of the BMP4 inhibitor GDF3 facilitated BMP4 to maintain pluripotency of mES cells [27]. This suggested that the isolated ES cells could maintain the long-term self-renewal ability *in vitro*.

As the vital component of ES cell medium, FBS is not an optimal supplement to maintain pluripotency of ES cells since it harbors some undefined factors which induce differentiation or apoptosis [5,7–9,12]. KSR, an artificial substitute for serum, had been proven preferable to FBS in maintaining pluripotency of mES cells in these mouse strains C57BL/6, BALB/cAJ, CBA/Ca and NOD [5–9,12]. However, some reports pointed out that partial replacement of KSR with FBS contributed better to increasing the efficiencies of ES cell isolation than complete replacement, when culturing embryos and initial ES cells [8,10,12]. Similarly, the results from our work indicated that in comparison with the sole use of FBS or KSR, recombined use of KSR and a small volume of FBS significantly contributed to increasing the efficiencies of ES cell isolation from hybrid embryos, and to formation of ES cell clones when being plated by the single ES cells. Also, we recommended that KSR and FBS would be supplemented together in the medium to efficiently establish ES cells when embryos and initial ES cells were cultured.

It has been proved that, in the presence of FBS or KSR, mES cells could be successfully isolated from the recalcitrant strains such as BALB/c, BALB/k, CBA/Ca and NOD by increasing LIF concentration [4,7,9,11,17]. The results from Peng *et al*.’s work on Kunming inbred mice [18] indicated that ES cells maintained undifferentiated *in vitro* in the feeder cell- and LIF-free condition by supplement with KSR, PD0325901 and CHIR99021, and contrarily lost the self-renewal capacity in the medium consisting of LIF and serum. Likewise, our present results indicated that ES cells from Kunming inbred mice could not propagate extendedly in the culture system composed of feeder cells, FBS, KSR and high concentration of LIF (100 ng/mL) since the vast majority of ICM outgrowths and subsequent ES cells quickly disappeared when being plated after the second digestion. This is in contrast with the fact that ES cells from hybrid embryos of Kunming and 129/Sv mice exhibited the powerful self-renewal capacity. Therefore, we presumed that establishment of ES cell lines from hybrid embryo of Kunming and 129/Sv mice, should be attributed to heterosis or 129/Sv genetic background, as the previous reports suggested [19,20], and LIF was insufficient to maintain the self-renewal ability of ES cells from Kunming inbred mice in the presence of serum or KSR, and feeder cells. Thus, it remains necessary to explore the genetic/epigenetic mechanism of hampering pluripotency of Kunming mouse-derive ES cells.

## 4. Experimental Section

### 4.1. Animals

Kunming mice (*Mus musculus* Km) and 129/Sv mice (purchased from the Laboratory Animal Centre in West-China Center of Medical Sciences, Sichuan University (Chengdu, China)) used in this study with ethical approval from the Animal Research Ethical Committee, were kept in temperature- and light-controlled rooms. Adult Kunming females injected by equine chorionic gonadotrophin (eCG) and human chorionic gonadotrophin (hCG) (purchased from Ningbo Second Harmone Factory, Ningbo, China), were mated with 129/Sv males or Kunming males overnight.

### 4.2. ES Cell Media

Three complete media for derivation and culture of ES cells, which were composed of high glucose DMEM (Invitrogen, Carlsbad, CA, USA), 20 ng/mL leukemia inhibitory factor (LIF) (Chemicon, Billerica, MA, USA), 1 mM nonessential amino acids (NEAA) (Invitrogen, Carlsbad, CA, USA), 0.1 mM β-mercaptoethanol (Sigma, Kanagawa, Japan), were supplemented with 15% Knockout serum replacement (KSR) (Invitrogen, Carlsbad, CA, USA) (*v*/*v*), 14% KSR plus 1% Fetal bovine serum (FBS) (Hyclone, Logan, UT, USA) (*v*/*v*), and 15% FBS (*v*/*v*), individually. In addition, LIF concentrations were increased to 100 ng/mL in the above media, then used to culture a part of Kunming mouse inbred embryos.

### 4.3. Isolation and Culture of ES Cells

Blastocysts for ES cell isolation, were flushed from uteri of the pregnant Kunming females, and cultured on the inactivated mouse embryonic fibroblasts (MEF) with 5 μg/mL mitomycin C (Sigma, Kanagawa, Japan). After embryos attached and formed ICM outgrowths with prominent nucleoli and dense morphology, ICM outgrowths were mechanically dissected into small cell clumps after treatment with trypsin (Invitrogen, Carlsbad, CA, USA) (0.5 mg/mL)/EDTA (Sigma, Kanagawa, Japan) (0.2 mg/mL), and individually plated on MEF feeder layers. The initial ES cells were subcultured by trypsinization in combination with mechanical dissection. When a large number of ES cell colonies appeared in the plates with 3.5 cm diameter, ES cell lines were ascertained.

### 4.4. Karyotype Analysis of ES Cells

Karyotype analysis of ES cells was performed as described previously [28]. Briefly, after cultured in feeder-free conditions for 24–36 h, ES cells were exposed to 10 μg/mL colchicine (Sigma, Kanagawa, Japan) for 1–2 h, and trypsinized into a single-cell suspension followed by suspension for 15 min in 0.075 mM KCl solution. Cells were repeatedly fixed in methanol/acetic acid mixture, then spread over slides and stained with Giemsa (Sigma, Kanagawa, Japan) solution. Karyotypes were observed and imaged under a Digital Microscopic Imaging System (Leica, DMIRB, Wetzlar, Gemany).

### 4.5. Genotype Analysis of mES Cells

To determine genetic source of the established mES cells, the microsatellite markers listed in Table 6, were examined by PCR for these samples including mES cells, tail tissues from Kunming female and 129/Sv male, and the F_1_ hybrid (KM × 129/Sv) embryos as controls. The microsatellite markers and their corresponding primer sequences were selected and obtained from Mouse Genome Database (http://www.informatics.jax.org) and the documents [29]. Genomic DNA of all samples were prepared according to instructions of the QIAamp DNA Mini Kit (Qiagen Co. Ltd., Shanghai, China). PCR reaction were carried out as follows: initial denaturation at 94 °C for 2 min, followed by 35 cycles of 15 s at 94 °C, 25 s at 60 °C, 35 s at 72 °C, and finally 10 min extension at 72 °C. PCR products were separated by electrophoresis on 4% agarose gel and imaged under the Gel Image System (Syngene Co. Ltd., Karnataka, UK). The genotypes of the tested samples were scored according to the traveling distance of PCR products of every microsatellite loci primers on the 4% agarose gel, with the shortest being given the genotype “a”, followed by “b”, “c”, and so on.

### 4.6. Analysis of Enzymes and Marker Molecules Specific for mES Cells

After being cultured for 48 h, ES cells were fixed with 4% formaldehyde and stained by alkaline phosphatase (AKP) or immunohistochemical methods. AKP staining was carried out as described previously [28], and the AKP-positive cells were stained as red or brown-red. Immunohistochemical staining was performed according to instructions of the SP-9000 General Immunohistochemical kit (Zhong-Shan Jinqiao Co. Ltd., Beijing, China). Briefly, cells were blocked with 10% goat serum plus 0.2% Triton X-100, then incubated at 4 °C overnight with primary antibody against Oct4 (1:100), SSEA-1 (1:100), SSEA-3 (1:100), SSEA-4 (1:100), Nanog (1:100) and telomerase (1:100) (all purchased from Chemicon Co. Ltd., Billerica, MA, USA), respectively. Subsequently, cells were exposed to TRITC-conjugated secondary antibodies (goat anti-mouse IgG), and would be dyed as red-brown or yellow-brown if positive.

RT-PCR was performed to examine expression of the genes *Oct*4, *Nanog*, *Gdf-*3 (a direct BMP4 inhibitor) and *GAPDH* as the inner control in ES cells. Total RNA was extracted according to instructions of the RNeasy Mini kit (Qiagen Co. Ltd., Shanghai, China) and reversely transcribed into cDNA using RevertAidTM First Strand Kits (Fermentas Co. Ltd., Waltham, MA, USA). PCR reaction were carried out as follows: 2 min denaturation at 94 °C, followed by 35 cycles of 30 s at 94 °C, 30 s at 55 °C, 60 s at 72 °C, and finally 10 min extension at 72 °C. PCR products were separated by electrophoresis and imaged under the Gel Image System (Syngene Co. Ltd., Shanghai, China). PCR primers for amplifying the genes *Oct*4, *Nanog*, *Gdf-*3 and *GAPDH* (as the inner control) listed in Table 7, were designed according to the corresponding gene sequences published in Gen*Bank*.

### 4.7. Pluripotency of mES Cells

Embryoid bodies (EBs) were prepared as described previously [27]. Briefly, ES cells were cultured in ES cell medium without the feeder layers for 24–28 h, then the cell clumps were mechanically separated and cultured in suspension in LIF-free medium containing 15% FBS until EBs formed. Subsequently, EBs were cultured in a gelatin-coated plate with LIF-free medium until various types of differentiated cells appeared. These cells were immunohistochemically stained with primary antibodies against the marker antigens specific for different germ layers, namely, α-fetoprotein (AFP) (1:50) for endoderm cells, α-Actin (1:100) for mesoderm cells, Nestin (1:100), β-Tubulin III (1:50) and GFAP (1:100) for ectoderm cells (all purchased from Chemicon Co. Ltd., Billerica, MA, USA), and would be dyed as red-brown or yellow-brown if positive. Meanwhile, osteoblasts from the mesoderm layer were stained as yellow-brown by Alizarin Red.

Moreover, EBs were harvested to examine expression of *Fgf*-5 and *Nf*-68 specific for ectoderm, *BraT* specific for mesoderm, *Afp* specific for endoderm, and *GAPDH* as the inner control by RT-PCR. PCR primers for amplifying the genes *Fgf*-5, *Nf*-68, *BraT* and *Afp* and *GAPDH* listed in Table 5, were designed according to the corresponding gene sequences published in Gen*Bank*.

ES cells (about 5 × 10^6^ cells/mouse) were subcutaneously injected into nude mice. After 4–6 weeks, the harvested teratomas were fixed in 4% formaldehyde to prepare paraffin sections. Sections were stained by hematoxylin/eosin (HE) and observed under a Digital Microscopic Imaging System (Olympus Co. Ltd., Tokyo, Japan).

### 4.8. Statistical Analysis

Statistical analyses were performed using SPSS11.5.0 Version (SPSS Inc., Chicago, IL, USA). An independent simple *t*-test was employed to analyze differences of attached rate (No. of attached blastocysts/No. of blastocysts cultured), ICM outgrowth rate (No. of ICM outgrowth/No. of blastocysts cultured) and establishing rate (No. of the established ES cell lines/No. of blastocysts cultured) between different groups. Statistical significance was taken as *p* < 0.05.

## 5. Conclusions

In summary, ES cell lines had been efficiently established from Kunming female mice by hybridization with 129/Sv male mice. Meanwhile KSR in conjunction with a small amount of FBS (1%) facilitated mES cell isolation, when blastocysts and primary ES cells were cultured. The results provided a new approach to ES cell isolation from Kunming mice and facilitated the exploration of the genetic/epigenetic mechanism related to Kunming mouse development.

## Figures and Tables

**Table 1.** The required time for embryo attachment in the different medium.

	No. of attached embryos	Time distribution for embryo attaching to the dishes (d)						

		2	3	4	5	6	7	
FBS	70	9	21	21	13	4	0	3.74 ± 1.10 ^a^
KSR	47	0	6	16	13	6	2	4.58 ± 1.05 ^b^

Note: In one vertical column, the data existed significantly different (*p* < 0.05) marked by the different letters, or no significantly different (*p* > 0.05) marked by the same letters. In the horizontal row of Tables 2–4, what the labels indicated were the same as here.

**Table 2.** Effects of fetal bovine serum (FBS) and knockout serum replacement (KSR) on establishment of embryonic stem (ES) cell lines.

	15% FBS	1% FBS + 14% KSR	15% KSR
No. of blastocysts plated	32	30	36
No. of attached embryos	22 (68.75%) ^a^	20 (66.67%) ^a^	15 (41.67%) ^b^
No. of ICM outgrowths	10 (31.25%) ^a^	18 (60.00%) ^b^	12 (33.33%) ^a^
No. of primary ES cell clones	4 (12.50%) ^a^	14 (46.67%) ^b^	5 (13.89%) ^a^
No. of the established ES cell lines	1 (3.13%) ^a^	14 (46.67%) ^b^	5 (13.89%) ^a^

**Table 3.** Effects of KSR and FBS on clone-forming efficiencies (%) of ES cells when single-cells were plated.

15% KSR	14% KSR + 1% FBS	15% FBS
14.2 (15/106) ^a^	35.7 (40/112) ^b^	41.3 (43/104) ^b^

**Table 4.** Comparison of ES cell establishment from hybrid embryos of Kunming and 129 mice and Kunming mouse inbred embryos.

	129 × KM	KM × KM
No. of blastocysts plated	98	391
No. of primary ES cell clones	23 (23.47%) ^a^	38 (9.72%) ^b^
No. of the established ES cell lines	20 (20.41%) ^a^	0 ^b^

**Table 5.** Genotype analysis of the established mES cells by microsatellite marker-based PCR.

Microsate-llite markers	KM females	129/Sv males	F_1_ embryos (KM × 129)	The tested mES cells
D1Mit15	c	-	c	c
D2Mit30	a	c	a/c	a/c
D4Mit54	-	a	-	-
D11Mit4	b	c	b/c	b/c
D11Mit167	b	b	b	b
D12Mit56	c	-	c	c
D14Mit129	a	b	a/b	a/b
D14Mit113	c	-	c	-
D17Mit36	-	c	c	c

Note: “a”, “b” and “c” refered to the bands of PCR products, and “-”represented no PCR products on the agarose gel.

**Table 6.** The primer sequences of microsatellite markers used in the present experiment.

Microsatellite markers	Forward primer	Reverse primer
D1Mit15	5′-TCCACAGAACTGTCCCTCAA-3′	5′-ATACACTCACACCACCCCGT-3′
D2Mit30	5′-TGCTGACCTGCTCAGCTG-3′	5′-AAATAACGTTTTCAATGAGATGG-3′
D4Mit54	5′-CTGCCATCCTGTAGTTTCACTG-3′	5′-ACCCCCACATATGTCTCCCT-3′
D11Mit4	5′-CAGTGGGTCATCAGTACAGCA-3′	5′-AAGCCAGCCCAGTCTTCATA-3′
D11Mit167	5′-TCGGATGCTAAGGAAATTGC-3′	5′-GACACTCAGTGTTGACCTCTGG-3′
D12Mit56	5′-GCTGTTTCACAGTCATTCATAACA-3′	5′-AACCTGCACAGGGTTTCCTT-3′
D14Mit129	5′-GGAGATGGTGGTAGAGGGGT-3′	5′-AGTTTGTGTGGTATGTGTAGGTGG-3′
D14Mit113	5′-TGCACAGGTTTTCCAATTTG-3′	5′-TGCTGTCTCTCCCCAAGC-3′
D17Mit36	5′-ATCTCACCAGTCCTTGTTTTCTG-3′	5′-CCCCAGAATTTATGTGGTGG-3′

**Table 7.** The primers and size of the amplified products.

Genes	Forward primer	Reverse primer	Product Size (bp)
*Oct*4	5′-TTCAGACTTCGCCTCCTCACCC-3′	5′-TTGTCGGCTTCCTCCACCCACTT-3′	600
*Nanog*	5′-TGGTGTCTTGCTCTTTCTGTGGG-3′	5′-GCACTTCATCCTTTGGTTTTG-3′	625
*Gdf*-3	5′-CCTTATCAACGGCTTCTGGCGC-3′	5′-CTCTAAGTGTAAGTCCAAGT-3′	606
*Fgf*-5	5′-CCTTGCTCTTCCTCATCTTCTGC-3′	5′-GAGCCATTGACTTTGCCATCCG-3′	319
*BraT*	5′-AAGGTGGCTGTTGGGTAGGGAGT-3′	5′-ATTGGGCGAGTCTGGGTGGATGT-3′	451
*Afp*	5′-ATCCTCCTGCTACATTTCGCTGC-3′	5′-TGAGCAGCCAAGGACAGAATG-3′	512
*TTR*	5′-ACTCTTCCTCCTTTGCCTCGCTG-3′	5′-GCAGGGGAGAAAAATGAGGAAAT-3′	592
*Nf*-68	5′-TTCTCCCCCGTTCTTCTCTCTAG-3′	5′-CTTCTCGTTAGTGGCGTCTTCC-3′	540
*GAPDH*	5′-CGGTGCTGAGTATGTCGTG-3′	5′-AGGTGGAAGAGTGGGAGTT-3′	616

Note: bp = base pairs.
